# Complete Mitochondrial Genomes of Two Toxin-Accumulated Nassariids (Neogastropoda: Nassariidae: *Nassarius*) and Their Implication for Phylogeny

**DOI:** 10.3390/ijms21103545

**Published:** 2020-05-17

**Authors:** Yi Yang, Hongyue Liu, Lu Qi, Lingfeng Kong, Qi Li

**Affiliations:** 1 Key Laboratory of Mariculture, Ministry of Education, Ocean University of China, Qingdao 266003, China; yiyangouc@outlook.com (Y.Y.); liuhongyue-mia@outlook.com (H.L.); qilu521@126.com (L.Q.); klfaly@ouc.edu.cn (L.K.); 2 Laboratory for Marine Fisheries Science and Food Production Processes, Qingdao National Laboratory for Marine Science and Technology, 1 Wenhai Road, Aoshanwei Town, Qingdao 266237, China

**Keywords:** *Nassarius glans*, *Nassarius siquijorensis*, mitochondrial genome, phylogeny, morphological synapomorphies, toxin

## Abstract

The Indo-Pacific nassariids (genus *Nassarius*) possesses the highest diversity within the family Nassariidae. However, the previous shell or radula-based classification of *Nassarius* is quite confusing due to the homoplasy of certain morphological characteristics. The toxin accumulators *Nassarius glans* and *Nassarius siquijorensis* are widely distributed in the subtidal regions of the Indo-Pacific Ocean. In spite of their biological significance, the phylogenetic positions of *N*. *glans* and *N*. *siquijorensis* are still undetermined. In the present study, the complete mitochondrial genomes of *N*. *glans* and *N*. *siquijorensis* were sequenced. The present mitochondrial genomes were 15,296 and 15,337 bp in length, respectively, showing negative AT skews and positive GC skews as well as a bias of AT rich on the heavy strand. They contained 13 protein coding genes, 22 transfer RNA genes, two ribosomal RNA genes, and several noncoding regions, and their gene order was identical to most caenogastropods. Based on the nucleotide sequences combining 13 protein coding genes and two rRNA genes, a well-supported phylogeny of *Nassarius* was reconstructed, and several morphological synapomorphies were observed corresponding to the phylogenetic framework. In addition, the sister group relationship between *N. variciferus* and the remaining toxin-accumulated nassariids was determined, suggesting that the phylogeny might be related to their diet. The divergence time estimation analysis revealed a correlation between speciation events of nassariids and glacial cycles during the Pliocene-Pleistocene epoch.

## 1. Introduction

Containing more than 600 extant species [1], the worldwide distributed mudsnail nassariids (Family Nassariidae Iredale, 1916) are an ecologically important group within Neogastropoda. As scavengers, nassariids are important in maintaining the balance of benthic ecosystem. Some nassariids are also useful for monitoring Tributyltin (TBT) pollution in marine environment [2]. Within Nassariidae, the cosmopolitan subfamily Nassariinae consists of five genera that correspond to five geographical clades. One of the five genera, called *Nassarius* (type genus of Nassariidae), is endemic to the Indo-Pacific Ocean [3] and possesses the highest diversity (about 300 species as valid [4]) within Nassariidae.

Traditional classification of *Nassarius* was mainly based on morphological characteristics [5]. However, the shell morphology-based taxonomy was revealed to be subjective and often contradicted molecular phylogenies [3,6,7]. On the other hand, previous *Nassarius* phylogenies, which were mainly based on short gene fragments (especially COI and/or 16S genes [8,9]), were poorly supported and often contradicted each other, suggesting the inadequate capacity of partial mitochondrial gene data for resolving the phylogeny of *Nassarius*. Using complete mitochondrial (mt) genomic sequences, Yang et al. [6] conducted the phylogeny of 10 common nassariids along the China Seas. The result revealed the advantages of utilizing complete mt genomes to address phylogenetic relationships within Nassariidae, as indicated in other caenogastropod groups [10,11,12,13]. Furthermore, the study called for the necessity of including more taxa into the reconstruction of the *Nassarius* phylogeny, which could provide a framework to understand the evolution of morphological and ecological traits.

In Asia, nassariids are consumed as seafood [7,14]. However, due to their ability to accumulate toxins (e.g., algal toxin or tetrodotoxin) [15,16], several poisoning incidents caused by the consumption of certain nassariids have been reported occasionally in some Asian countries [17,18,19]. Recent studies found that the toxicity varied in species [20,21]. For example, most marine nassariids are toxin accumulators in addition to *Nassarius variciferus* and *Reticunassa festiva* which could not accumulate toxins. Among those toxin accumulators, *N. siquijorensis* and *N. glans* are widely distributed in the subtidal regions of the Indo-Pacific Ocean. The ecology and toxicology of *N. siquijorensis* and *N. glans* have been the subjects of numerous studies [20,22]. In spite of the biological significance, the classification and phylogenetic positions of *N. siquijorensis* and *N. glans* within *Nassarius* are still controversial. For example, the sister group of *N. siquijorensis* and *N. nodifer* revealed by Chen and Zhang [8] was not recovered by Pu et al. [23], although both studies were poorly supported.

In the present study, the complete mitochondrial genomes of *N. siquijorensis* and *N. glans* were sequenced and analyzed together with those of other nassariids publishes before (Table 1). Our aims were (1) to confirm the phylogenetic positions of *N. siquijorensis* and *N. glans* within *Nassarius*; (2) to test the contribution of different data sets of mitochondrial genomes for assessing phylogenetic relationships in *Nassarius*; and (3) to date major cladogenetic events within *Nassarius*.

## 2. Results and Discussion

### 2.1. Genome Structure, Organization, and Composition

**Figure 1:**
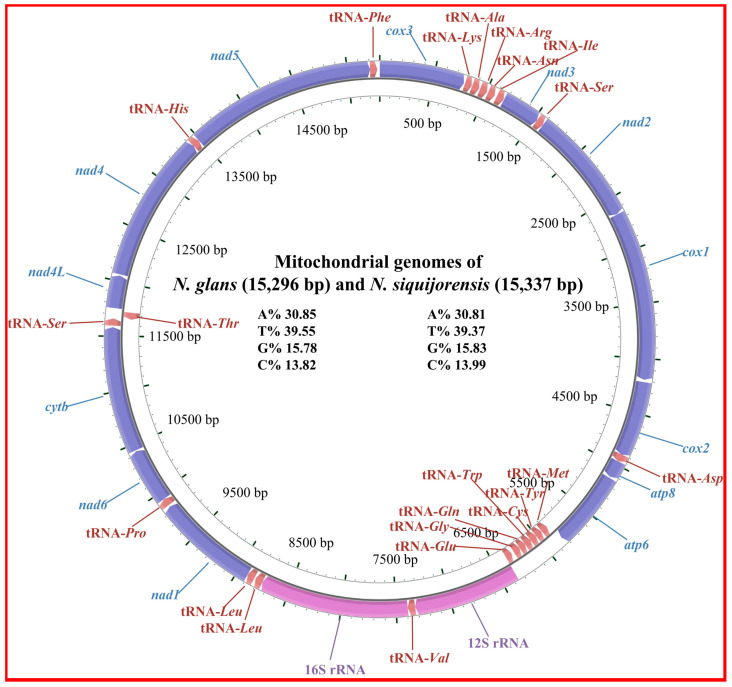
Gene map of the mt genomes of *N. glans* and *N. siquijorensis*.

The characteristics of the two mt genomes in the present study were similar to those of the other nassariids reported before [6,24,25,26] in terms of genome organization and nucleotide composition (Figure 1). The mt genomes of *N. siquijorensis* and *N. glans* are 15,337 and 15,296 bp in length, respectively. Both of them encode for 13 protein-coding genes (PCGs), 22 transfer RNA (tRNA) genes. and two ribosomal RNA (rRNA) genes, with 8 tRNA genes encoding in the minor strand while the others encode in the major strand (Table 2). 

The AT contents and skew statistics are shown in Table 3, indicating a high A + T bias. The result also showed that the nucleotide compositions were skewed from A towards T but insignificantly skewed from C towards G. This strand asymmetry has been observed in other gastropod taxa, such as Vetigastropoda [27], Heterostropha [28], Neritimorpha [29], and Patellogastropoda [30], where T was more than A and C was less than G on the heavy strands. In addition, the heavy strands of mammalian mt genomes were also found to be GT rich [31] whereas both strands of arthropod mt genomes were AT rich [32,33]. The mechanism of asymmetry of the heavy strand was attributed to the hydrolytic deaminations of adenine and cytosine (leading to the mutations from adenine to guanine and from cytosine to thymine) during replication and transcription as well as during transcription but to a lesser extent [32].

### 2.2. PCGs, rRNA, and tRNA Genes

The AT content, AT skew, and GC skew of the PCGs were also similar in the mt genomes of *N. siquijorensis* and *N. glans* (Table 3). In the PCGs, all AT skews were negative while most GC skews were positive. It was notable that a significantly negative GC skew was found in the *nad5* genes of both species. This reversed bias might be attributed to the effect of the control region [32], which was located between tRNA-*Phe* and *cox3* genes. The average A + T content values of PCGs were 69.73% and 69.78% in *N. glans* and *N. siquijorensis*, respectively, and those proportions at the first, second, and third codon positions were 61.28% and 61.17%, 62.88% and 62.85%, and 85.03% and 85.33%, respectively, indicating a strong A + T bias at the third codon position as reported in other invertebrate mt genomes (e.g., Cnidaria [34] and Arthropoda [35]). Almost all PCGs of the 2 mt genomes started with the conventional initiation codon ATG, except for the *nad4* of *N. glans* which began with ATA (Table 2).

**Figure 2:**
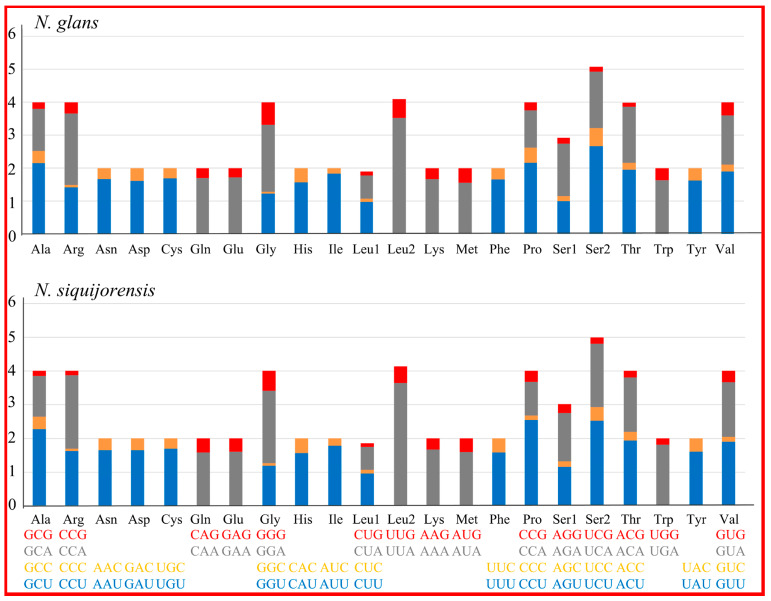
Relative Synonymous Codon Usage (RSCU) of mitochondrial genomes for *N. glans* and *N. siquijorensis*.

As for the termination codons, all PCGs ended with the complete stop codons (TAA, n = 17; TAG, n = 9). Codon usage of PCGs was presented in Table 4. Both 2 mt genomes had 3729 PCG codons (excluding the stop codons), among which the most frequently used one was UUA (leucine) while the least chosen codon was CGC (arginine) (Table 4 and Figure 2). On the other hand, the most encoded amino acid was leucine while the least encoded one was cysteine. Considerable synonymous codon usage bias was observed in both mt genomes, with a total number of 20 codons being used more frequently than others (Table 4). Those preferred codons were detected all ending in A or U and therefore resulted in a strong A + T bias at the third codon position. Previous studies revealed that the synonymous codon usage bias might be caused by mutational bias alone or by both mutation bias and natural selection [36]. The four most used codons UUA (leucine), AUU (isoleucine), UUU (phenylalanine) and AUA (Methionine) observed here (Figure 2 and Table 4) also fit with some other metazoan taxa, such as in Annelida [37] and Nematoda [38].

**Figure 3:**
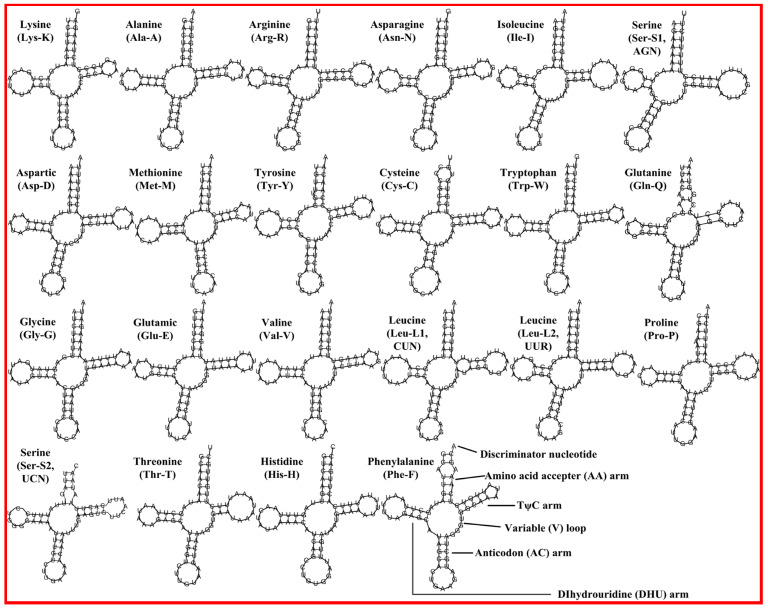
Inferred secondary structures of 22 tRNAs from *N. glans*: The tRNAs are labelled with their corresponding amino acids. Structural elements in tRNA arms and loops are illustrated as for trnF.

The lengths of the tRNA genes between *N. glans* and *N. siquijorensis* were almost identical, ranging from 57 (*trnS*-UCN of *N. siquijorensis*) to 71 bp (*trnL*-CUN of *N. glans*). All of the tRNA genes could be folded into typical clover-leaf secondary structures except for the *trnS*-AGN in both mt genomes due to the missing of dihydrouracil (DHU) arms (Figure 3). The lack of DHU arm in *trnS*-AGN was quite common in metazoan mt genomes [39]. The average AT contents of tRNA genes were 68.90% and 68.73% in *N. glans* and *N. siquijorensis*, respectively, and the tRNA genes were neither substantially AT nor GC skewed (Table 3).

The 12S rRNA genes of *N. glans* and *N. siquijorensis* were 964 and 962 bp in length, with AT contents of 68.80% and 67.64%, respectively, while the 16S rRNA genes were 1361 and 1359 bp, with AT contents of 73.82% and 72.84% in *N. glans* and *N. siquijorensis*, respectively. Unlike the tRNA genes, the rRNA genes showed a weakly positive AT skew and strongly positive GC skew (Table 3).

### 2.3. Phylogenetic Relationship

Phylogenetic analysis was conducted using two data sets. Based on the Bayesian information criterion (BIC) [40], the best partition scheme for PCGs at nucleotide level was the one combining all these genes but analyzing the three codons separately. The best substitution models were GTR + I + G for all three codon positions. At the amino acid level, the best partition scheme for PCGs was the one combining genes by subunits. The best substitution models were MTART + I for *atp*, *cob*, and *cox*, and MTMAM + I + G + F for *nad*. For the rRNA genes, the best partition scheme was combining 12S and 16S rRNA genes together, with GTR + I + G as the best substitution model.

**Figure 4:**
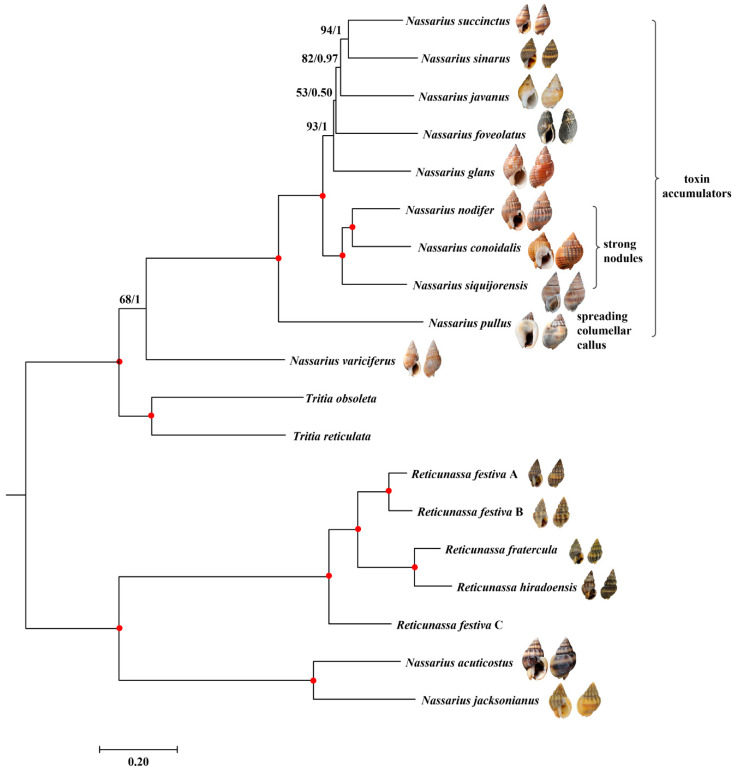
Phylogenetic relationship of *Nassarius* based on nucleotide sequences of 13 mitochondrial PCGs and 2 rRNA genes: The reconstructed Bayesian inference (BI) phylogram (generated by Mybayes program) using “*Nassarius*” *acuticostus*, “*Nassarius*” *jacksonianus*, and *Reticunassa* as outgroup is shown. The first number at each node is bootstrap proportion (BP) of maximum likelihood (ML) analyses, and the second number is Bayesian posterior probability (PP). Nodal with maximum statistical supports (BP = 100; PP = 1) is marked with a solid red circle.

The final matrix of the first data set (nucleotide sequences of 13 PCGs and rRNA genes) was 13,277 bp in length. Both maximum likelihood (ML) [41] and Bayesian inference (BI) [42] arrived at almost identical topologies (Figure 4 and Figure S1), and most nodes received high support values, except for one internal node that connected *N. foveolatus* and *N. javanus* + (*N. sinarus* + *N. succinctus*). The second data set (amino acid sequences of PCGs plus nucleotide sequences of rRNA genes) was 5821 positions in length. However, the phylogenetic analysis based on the second data set was not well resolved due either to the relatively lower statistical support values or to the contradictory topologies reconstructed using ML and BI methods (Figure S1). Compared with the first data set, the second one had fewer genetic sites and the amino acid sequences appeared to be more conserved than the nucleotide sequences, as indicated by the short branch lengths of the phylogenetic trees (Figure S2). As a result, the present study shows that the combining the nucleotide sequences of 13 PCGs and two rRNA genes could provide enough variances for the divergence within genus *Nassarius*.

Except for *N. variciferus*, *N. foveolatus*, and *N. sinarus*, all the remaining species analyzed in the present study were also included in the phylogeny conducted by Galindo et al. [3] (Note that *N. nodifer* here is synonymized as *N. hepaticus* in Galindo et al. [3]). Compared with the most comprehensive phylogenetic study on Nassariidae published thus far [3], the present phylogeny showed different topologies. In the present tree, *N. siquijorensis* was revealed as the sister group of *N. nodifer* + *N. conoidalis* while *N. glans* clustered with *N. foveolatus* + (*N. javanus* + (*N. succinctus* + *N. sinarus*)). On contrast, *N. glans* showed a closer relationship with *N. succinctus* while *N. siquijorensis* was more related to *N. conoidalis* + (*N. javanus* + *N. nodifer*) in the nassariid tree of Galindo et al. [3]. Compared with previous phylogenies which were not well supported, the present study could improve the resolution of phylogenetic relationship of these targeted nassariids. However, it is also shown in the tree (Figure 4) that some internal branches connecting the nodes with relatively lower statistical support are rather short, which indicates a rapid radiation [43]. Consequently, achieving high statistical support of these phylogenetic relationships will be challenging [44]. In the future, it is necessary to sequence more mt genomes of taxa within this clade as well as to develop new molecular markers (e.g., exon-capture based on transcriptomic data [45,46]) in order to resolve the rapid radiation.

In the present phylogeny (Figure 4), *N. variciferus* was the first species branching off. The distant phylogenetic relationship between *N. variciferus* and other nassariids has also been revealed in previous studies [6,7]. Based on the morphology of shell characteristic, *N. variciferus* was previously classified into subgenus *Varicinassa*, which was defined as a single-species group with prominent angulate varices on whorls [5]. However, the presence of varices appears to be a morphological phenotype because individuals of *N. variciferus* without varices have also been found [7]. According to previous records, almost all *Nassarius* species were toxin accumulators except for *N. variciferus* (Table 1). It is inferred that nassariid toxins, which were recognised as algal toxin or tetrodotoxin, could not be produced by nassariids themselves, but might be from the environment (probably via the food chains [47]). Therefore, the differences on toxicity may reflect different food preferences. However, further studies concerning the components and sources of nassariid toxins as well as the anatomical characteristics of nassariid digestive systems are needed to support the present hypothesis.

**Figure 5:**
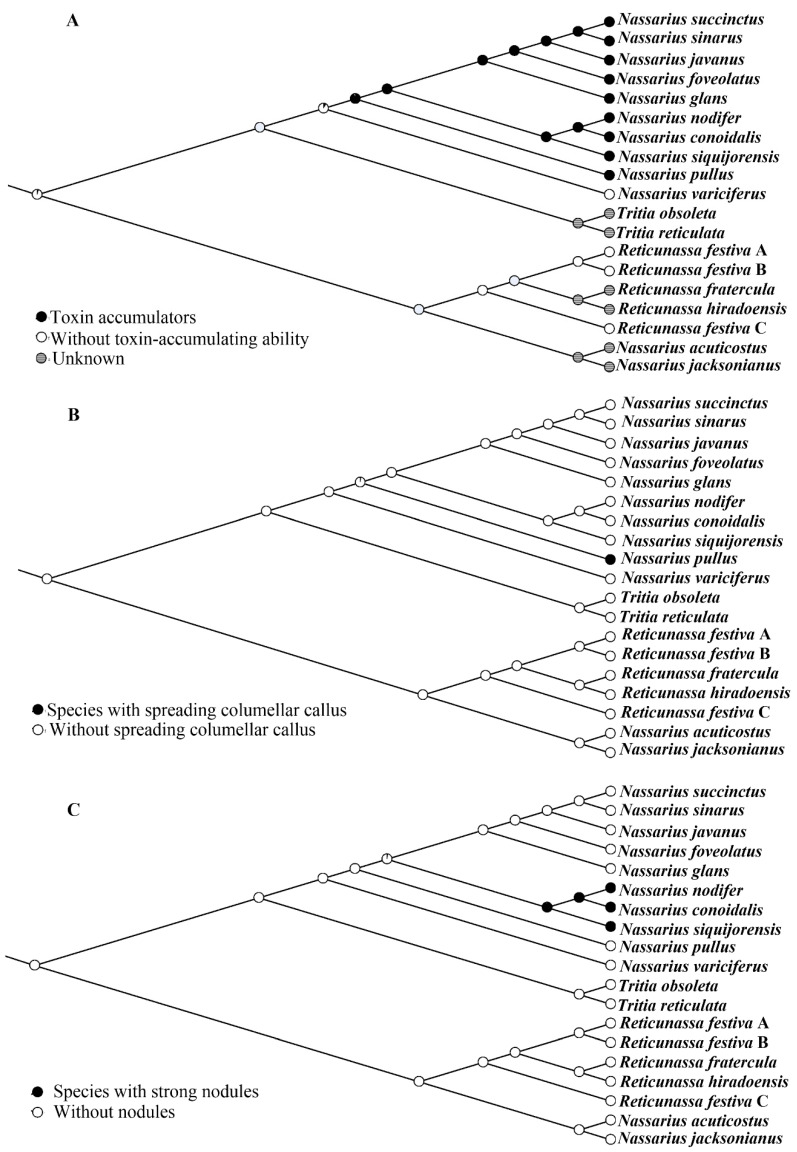
Ancestral character state reconstruction of three characteristics using the maximum likelihood model in Mesquite: Pie charts represent the degree of support at every node.

In addition to *N. variciferus*, the remaining nassariids form into three lineages, including the single-species *N. pullus*; the clade grouped by *N. nodifer*, *N. conoidalis*, and *N. siquijorensis*; and the group formed by all the others (Figure 4). The distant phylogenetic relationship between *N. pullus* and other nassariids is also supported by the morphological characteristics. The most significant trait of *N. pullus* is the spreading columellar callus which was used to define subgenus *Plicarcularia* [5]. However, it is insufficient to determine whether this characteristic could unite other members in phylogeny since only one species of *Plicarcularia* is included here. In the second lineage, one unique trait that connects *N. nodifer*, *N. conoidalis*, and *N. siquijorensis* has also been observed in the present study. Morphologically, all these three species possess strong nodules formed by the axial and spiral ribs. In previous classification [5], most members in subgenus *Niotha* have these morphological traits. Even though *N. siquijorensis* possesses this characteristic, it was incorrectly classified into subgenus *Zeuxis*, which was a not particularly well-defined group, and its value from a taxonomic viewpoint is doubtful. Based on the present phylogeny, *N. siquijorensis* should be reassigned from *Zeuxis* to *Niotha*, and the existence of nodules on shell surface should be considered as one of the most important synapomorphies (Figure 5) to define this group. According to a previous study [23], a total of 23 individuals of *N. siquijorensis* collected in five sites along Guangdong Province, China were recovered to form two different clades in the *cox1*-based phylogeny, indicating that *N. siquijorensis* possessed cryptic species. Geographically, the sampling site of *N. siquijorensis* in our study did not correspond to any of the five sites in Pu et al. [23]. Although the phylogenetic position of *N. siquijorensis* was determined here, the circumscription of *N. siquijorensis* still remains unclear. Further studies with a high sampling coverage along its distribution range are needed to determine its diversity and to define the status of the *N. siquijorensis* complex. In the third clade, the other five species were previously assigned into three subgenera (*Zeuxis*, *Telaseo*, and *Alectrion*). Nevertheless, no significant morphological characteristic was found corresponding to the current phylogeny due to the limited species number. Consequently, the present study calls for the inclusion of more mitogenomic data into the reconstruction of *Nassarius* phylogeny, which is useful for the reestablishment of *Nassarius* taxonomy.

### 2.4. Divergence Times

**Figure 6:**
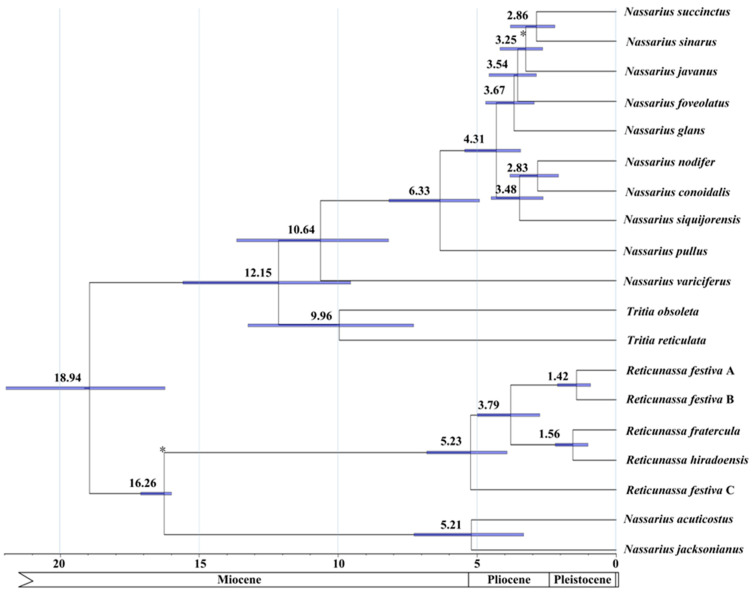
Divergences time estimations for the Nassariidae using Bayesian relaxed dating methods (BEAST) based on the nucleotide sequences of 13 PCGs: Dates (and credibility intervals) are in millions of years, and horizontal bars represent 95% credibility intervals of relevant nodes. Calibration constraints are indicated with an asterisk on the corresponding nodes.

Major cladogenetic events within *Nassarius* were dated using an uncorrelated relaxed molecular clock model, which was calibrated using two fossils. The first event of diversification within the crown group of *Nassarius* was estimated at a mean of 10.6 (13.7–8.2, credible interval) million years ago (Mya), separating *N. variciferus* and the remaining nassariids which have the ability to accumulate toxins. The branching of *N. pullus* and the other two major lineages was estimated to have occurred at 6.3 (8.2–4.8) Mya. Finally, the speciation events leading to the other extant *Nassarius* were estimated to have occurred between 4.3–2.8 (5.3–2.2) Mya (Figure 6). However, the estimated divergence times of previous studies were much earlier. For example, the divergence time between *R. festiva* A and *R. festiva* B was estimated to 5.05 Mya [6]; the speciation between *N. hepaticus* (synonymized as *N. nodifer*) and *N. conoidalis* was dated to about 12 Mya [3]. These results (earlier estimated divergence times) might be attributed to the employment of older fossils, such as using the fossil time of oldest *Nassarius* from Europe (28–23 Mya) as the divergence between *Nassarius* and *Tritia* [3,6]. It is shown here that most speciation events happened during Pliocene and Pleistocene, corresponding with the fossil records of most extant *Nassarius* in the Paleobiology Database [48]. In addition, previous studies have revealed that *R. festiva* A and *R. festiva* B were closely related species [26] and that their diversification time might date back to Pleistocene [49]. The estimated divergence time between *R. festiva* A and *R. festiva* B (1.42 Mya) shown here also falls into Pleistocene, supporting the hypothesis that the speciation might be caused by the third cooling event during the early Pleistocene (2.2–1.0 Mya) [50]. Furthermore, the divergence time between *Nassarius* and *Tritia* revealed here (12.15 Mya) is also supported by the fossil time of the oldest *Tritia* (about 13 Mya) recorded in the Paleobiology Database [48].The ancestral character state reconstruction analysis (Figure 5) suggests that *Nassarius* may have originated from an ancestor without toxin-accumulating ability. The divergence time between *N. variciferus* and the remaining toxin accumulators is dated to Miocene, corresponding to the period when nassariids diversified dramatically [51]. The divergence time of *N. pullus* also falls in this epoch. In the last 5.3 Mya, there were three major cooling periods discovered in the Western Pacific: the early Pliocene (4.7–3.5 Mya), the late Pliocene (3.1–2.7 Mya), and the early Pleistocene (2.2–1.0 Mya) [50]. The divergence times of the remaining nassariids broadly fall in the three cooling periods, suggesting a correlation between speciation events and glacial cycles. The rapid radiation of certain nassariids may be attributed to the first cooling event.

## 3. Materials and Method

### 3.1. Samples and DNA Extraction

Specimens of *N. siquijorensis* and *N. glans* were collected in the same local market in Wenchang, Hainan Province, China (19°32′6″N; 110°49′13″E). Samples were then deposited in 95% ethanol.

Genomic DNA was extracted from small pieces of foot tissue using TIANamp Marine Animals DNA Kit (TIANGEN Biotech Beijing Co. Ltd.), according to the instructions, and visualized on 1.0% agarose gel. Only one specimen of each species was used for DNA extraction. The DNA concentration was measured on the Nanodrop 2000.

### 3.2. DNA Sequencing and Genome Assembly

Genomic DNA of *N. siquijorensis* and *N. glans* were submitted to Novogene Company (Beijing, China) for library construction and high-throughput sequencing. Two sequencing libraries with average insert sizes of approximately 300 bp were prepared and then sequenced as 150 bp paired-end runs on the Illumina HiSeq 2000 platform. Finally, about 8 Gb of raw data were generated for each library.

After removing the contaminated reads, low-quality sequences, and adapters (following Reference [52]), the clean reads of 2 species were de novo assembled separately using SPAdes 3.12.0 [53].

### 3.3. Gene Annotation and Sequence Analysis

The newly determined mt genomes were annotated with Geneious Prime 2019.0.3 [54], using the previously published *Nassarius* mt genomes [6,26] as references. Annotations of 13 PCGs were defined by setting a limit of 75% nucleotide identity to previously published nassariid mt genomes (Table 1) in Geneious Prime 2019.0.3 and corroborated using MITOS Webserver [55] by setting the Invertebrate Mitochondrial genetic code. The secondary structure of tRNA genes was generated by MITOS Webserver and modified using Microsoft Visio 2010 according to the tRNA structure identified by tRNA scan-SE1.21 [56] and ARWEN [57]. The rRNA genes were identified by comparing with other nassariid mt genomes, and their boundaries were assumed to be between the adjacent genes.

Codon usage of PCGs; the A + T content values; and nucleotide composition of the mt genomes, PCGs, rRNA, and tRNA genes were estimated using MEGA 7 [58]. The base skew values for a given strand was calculated as AT skew = (A − T)/(A + T) and GC skew = (G − C)/(G + C) [59]. The mt gene map was generated using CGView [60].

A total of 19 taxa was included in the phylogenetic analysis. Seven species, including five *Reticunassa*, “*Nassarius*” *acuticostus*, and *“Nassarius” jacksonianus,* were selected as outgroup taxa [6]. The 13 PCGs were aligned separately using Translator X [61], according to the Invertebrate Mitochondrial genetic code. Nucleotide sequences of the rRNA genes were aligned separately using MAFFT v7 [62] with default parameters. Ambiguously aligned positions were removed using Gblocks v.0.91b [63]. Finally, the different single alignments were concatenated into the two data sets in Geneious Prime 2019.0.3. Two data sets were constructed: 1. the nucleotide sequences of 13 PCGs and 2 rRNA genes and 2. the amino acid sequences of 13 PCGs and the nucleotide sequences of 2 rRNA genes.

### 3.4. Phylogenetic Analysis

Phylogenetic analyses were inferred using ML and BI. ML analyses were carried out using software RAxML v. 8.2.1 [64] with the rapid hill-climbing algorithm and 10,000 bootstrap pseudoreplicates (BP). BI analyses were performed with MrBayes v.3.1.2 [65], running four simultaneous Monte Carlo Markov chains (MCMC) for 10,000,000 generations, sampling every 1000 generations, and discarding the first 25% generations as burn-in. Parameter convergence was achieved within 10 million generations, and the standard deviation of split frequencies was less than 0.01. All parameters were checked with Tracer v. 1.6 [66], and the effective sample size (ESS) was more than 200. The resulting phylogenetic trees were visualized in FigTree v1.4.2 [67]. BI analyses based on nucleotide sequences of PCGs only with site-heterogeneous mixture models were conducted in PhyloBayes MPI v.1.7b [68], using the CAT-GTR model and discarding constant sites (“-dc” option) on the CIPRES webserver [69]. Parameters to convergence were set according to Uribe et al. [70].

The best partition schemes and best-fit substitution models for the two data sets were determined by PartitionFinder 2 [71] under the BIC. For the PCGs analyzed at both nucleotide and amino acid levels, the partitions tested were all genes combined; all genes separated (except *atp6*-*atp8* and *nad4*-*nad4L*); and genes grouped by subunits (*atp*, *cob*, *cox*, and *nad*). At the nucleotide level, these three partition schemes were tested considering separately the three codon positions. The rRNA genes were analysed with two different schemes (genes grouped or separated).

### 3.5. Divergence Time Estimation

The divergence times among *Nassarius* species were estimated based on the protein-coding genes (at nucleotide level) only, using an uncorrelated, lognormal relaxed molecular clock model in BEAST v.1.10.4 [72]. For the tree prior, a Yule process of speciation was employed. The partitions selected by PartitionFinder 2 (see above) were applied. The final Markov chain was run twice for 100 million generations, sampling every 10,000 generations, and the first 10% samples was discarded as burn-in according to the convergence of chains checked with Tracer v. 1.6. The effective sample size of all the parameters was above 200.

The posterior distribution of the estimated divergence times was obtained by specifying two calibration points as priors for divergence times of the corresponding splits. The first calibration point was set for the divergence of *Reticunassa* (16.0 Mya, [73]), with a minimum of 16 Mya and a 95% upper limit of 17.5 Mya (exponential distribution, offset: 16.0; mean: 0.5). A second calibration point was set based on the fossil time of *N. dorsatus* (3.6–2.6 Mya, [74]) that likely corresponded to the divergence time between *N. javanus* and *N. succinctus* + *N. sinarus* [75].The minimum and 95% upper limits were set to 2.6 and 3.6 Mya, respectively (lognormal distribution, offset: 2.6; mean: 0.4; standard deviation: 0.4).

### 3.6. Ancestral Character State Reconstruction

The evolution of three traits (Figure 5) was analyzed by reconstruction of the ancestral character states. These characteristics were assessed with Mesquite v3.6.1 [76] using Tracing Character History option and ML approach and mapped over the phylogenetic tree generated by ML and BI analyses.

## 4. Conclusions

The complete mitochondrial genomes of *N. glans* and *N. siquijorensis* were sequenced in the present study. Containing 13 PCGs, 22 tRNA genes, two rRNA genes, and several noncoding regions, the two mt genomes showed a similar pattern with respect to genome size, gene order, and nucleotide composition compared with those of other nassariids reported before. The data set combining nucleotide sequences of 13 PCGs and two rRNA genes was revealed to be most useful for phylogenetic reconstruction of *Nassarius*. The phylogenetic positions of *N. glans* and *N. siquijorensis* were well determined, and several morphological synapomorphies were observed corresponding to the present phylogeny. The present study also proposed a hypothesis that the phylogeny of *Nassarius* was related to their diet, based on the sister group relationship between the *N. variciferus* and the remaining toxin accumulators. A correlation between nassariid speciation events and Pliocene-Pleistocene glacial cycles was presumed. Our results indicate that complete mt genomes would be a promising tool to reconstruct a robust phylogeny of *Nassarius* with the inclusion of more taxa in the future. This approach could be complemented with the development of nuclear markers, which could be useful for eliminating the influence of rapid radiations.

## Figures and Tables

**Table 1:** List of the mt genomes analysed in the present study.

**New mt genomes**				
**Species**	**Length (bp)**	**Sampling time**	**Accession no.**	**Toxicity Record**
*Nassarius siquijorensis*	15,337	April, 2017	MN983149	Toxin accumulators [19]
*Nassarius glans*	15,296	October, 2018	MN983150	Toxin accumulators [20]
			
**Genbank mt genome**				
**Species**	**Length (bp)**	**Accession no.**	**Reference**	**Toxicity Record**
*Tritia obsoleta*	15,263	DQ238598	[24]	Unknown
*Tritia reticulata*	15,271	EU827201	[25]	Unknown
*Nassarius variciferus*	15,269	KM603509	[26]	Not toxin accumulators [21]
*Nassarius succinctus*	15,329	KT768016	[6]	Toxin accumulators [19]
*Nassarius nodifer*	15,337	KT818617	[6]	Toxin accumulators [21]
*Nassarius conoidalis*	15,332	KT826694	[6]	Toxin accumulators [21]
*Nassarius pullus*	15,278	KT900947	[6]	Toxin accumulators [17]
*Nassarius sinarus*	15,325	MH346208	[6]	Toxin accumulators [19]
*Nassarius foveolatus*	15,343	MH346209	[6]	Toxin accumulators [21]
*Nassarius javanus*	15,325	MH346210	[6]	Toxin accumulators [21]
*Reticunassa fratercula*	15,174	KT826695	[26]	Unknown
*Reticunassa hiradoensis*	15,194	MG744569	[26]	Unknown
*Reticunassa festiva*-A	15,195	KT735055	[26]	Not toxin accumulators [21]
*Reticunassa festiva*-B	15,194	MF148855	[26]	Not toxin accumulators [21]
*Reticunassa festiva*-C	15,172	MG744570	[26]	Not toxin accumulators [21]
*Nassarius jacksonianus*	15,234	MH346212	[6]	Unknown
*Nassarius acuticostus*	15,240	MH346211	[6]	Unknown

**Table 2:** Gene annotations of the complete mt genome of *N. glans* and *N. siquijorensis*: Identical values are shown only once.

*N. glans*/*N. siquijorensis*						
Gene	Strand	Location (bp)	Size (bp)	Start Codon	Stop Codon	Intergenic Nucleotides (bp)
*cox3*	H	1–780/1–780	780	ATG	TAA	10/9
tRNA-*Lys*	H	791–855/790–856	65/67			4/3
tRNA-*Ala*	H	860–927/860–928	68/69			9
tRNA-*Arg*	H	937–1005/938–1006	69			11/17
tRNA-*Asn*	H	1017–1082/1024–1092	66/69			20/18
tRNA-*Ile*	H	1103–1171/1111–1179	69			3
*nad3*	H	1175–1528/1183–1536	354	ATG	TAA/TAG	0
tRNA-*Ser*	H	1529–1596/1537–1604	68			0
*nad2*	H	1597–2652/1605–2660	1056	ATG	TAA	3/0
*cox1*	H	2656–4191/2661–4196	1536	ATG	TAA/TAG	21/23
*cox2*	H	4213–4899/4220–4906	687	ATG	TAA	−2
tRNA-*Asp*	H	4898–4965/4905–4972	68			1
*atp8* *atp6* tRNA-*Met*	H H L	4967–5125/4974–5132 5136–5831/5141–5836 5865–5931/5870–5935	159 696 67/66	ATG ATG	TAA TAG/TAA	10/8 33 4/5
tRNA-*Tyr*	L	5936–6001/5941–6007	66/67			1
tRNA-*Cys*	L	6003–6069/6009–6072	67/64			0
tRNA-*Trp*	L	6070–6136/6073–6139	67			−2
tRNA-*Gln*	L	6135–6201/6138–6204	67			0
tRNA-*Gly*	L	6202–6269/6205–6271	68/67			25/20
tRNA-*Glu*	L	6295–6361/6292–6358	67			0
12s tRNA-*Val* 16s	H H H	6362–7325/6359–7320 7326–7393/7321–7389 7394–8754/7390–8748	964/962 68/69 1361/1359			0 0 0
tRNA-*Leu*	H	8755–8825/8749–8817	71/69			11/7
tRNA-*Leu*	H	8837–8905/8825–8893	69			0
*nad1*	H	8906–9847/8894–9835	942	ATG	TAA	15/14
tRNA-*Pro*	H	9863–9931/9850–9918	69			1
*nad6*	H	9933–10433/9920–10420	501	ATG	TAA	8
*cytb*	H	10442–11581/10429–11568	1140	ATG	TAA	1/16
tRNA-*Ser*	H	11583–11643/11585–11641	61/57			17/8
tRNA-*Thr*	L	11661–11728/11650–11718	68/69			26/35
*nad4L*	H	11755–12051/11754–12050	297	ATG	TAG	11
*nad4*	H	12063–13418/12062–13417	1356	ATA/ATG	TAG	−1
tRNA-*His*	H	13418–13485/13417–13484	68			1
*nad5*	H	13487–15208/13486–15207	1722	ATG	TAG	−1
tRNA-*Phe*	H	15208–15274/15207–15274	67/68			22/63

**Table 3:** List of AT content, AT skew, and GC skew of *N. glans* (*Ngla*) and *N. siquijorensis* (*Nsiq*).

	(A + T)%		AT Skew		GC Skew	
	*Ngla*	*Nsiq*	*Ngla*	*Nsiq*	*Ngla*	*Nsiq*
Genome	70.40	70.20	−0.12	−0.12	0.07	0.06
PCGs	69.73	69.78	−0.18	−0.18	0.05	0.04
PCGs 1th	61.28	61.17	−0.10	−0.10	0.25	0.25
PCGs 2th	62.88	62.85	−0.41	−0.41	−0.15	−0.14
PCGs 3th	85.03	85.33	−0.07	−0.07	0.02	−0.02
*rrnS*	68.80	67.64	0.09	0.09	0.17	0.15
*rrnL*	73.82	72.84	0.03	0.04	0.15	0.15
tRNAs	68.90	68.73	−0.03	−0.02	0.07	0.06
*atp6*	71.14	71.72	−0.26	−0.23	−0.04	−0.05
*atp8*	75.64	74.36	−0.08	−0.07	0.05	0
*cob*	68.34	68.43	−0.20	−0.22	−0.04	0
*cox1*	66.60	66.60	−0.19	−0.19	0.08	0.08
*cox2*	67.98	69.01	−0.12	−0.13	0.13	0.15
*cox3*	64.22	64.86	−0.25	−0.24	0.22	0.16
*nad1*	68.16	68.48	−0.22	−0.22	0.10	0.07
*nad2*	72.08	72.27	−0.20	−0.20	0.23	0.25
*nad3*	69.80	70.37	−0.27	−0.27	0.23	0.19
*nad4*	71.99	71.40	−0.16	−0.13	−0.04	−0.06
*nad4L*	72.11	73.13	−0.14	−0.15	0.10	0.11
*nad5*	70.80	70.80	−0.11	−0.11	−0.14	−0.14
*nad6*	74.90	72.89	−0.22	−0.22	0.04	0.08

**Table 4:** Codon and relative synonymous codon usage (RSCU) of 13 PCGs in the mt genomes of *N. glans* (*Ngla*) and *N. siquijorensis* (*Nsiq*).

Amino	Codon	Count (RSCU)		Amino	Codon	Count (RSCU)	
Acid		*Ngla*	*Nsiq*	Acid		*Ngla*	*Nsiq*
Phe	UUU	**263 (1.66) ^a^**	**249 (1.59)**	Ala	GCU	**130 (2.15)**	**140 (2.27)**
	UUC	53 (0.34)	65 (0.41)		GCC	23 (0.38)	23 (0.37)
Leu	UUA	**345 (3.53)**	**359 (3.64)**		GCA	77 (1.27)	75 (1.21)
	UUG	56 (0.57)	49 (0.5)		GCG	12 (0.2)	9 (0.15)
	CUU	96 (0.98)	95 (0.96)	Gly	GGU	76 (1.23)	73 (1.19)
	CUC	10 (0.1)	11 (0.11)		GGC	4 (0.06)	5 (0.08)
	CUA	68 (0.7)	67 (0.68)		GGA	**126 (2.03)**	**131 (2.14)**
	CUG	12 (0.12)	11 (0.11)		GGG	42 (0.68)	36 (0.59)
Ile	AUU	**286 (1.83)**	**278 (1.78)**	Arg	CGU	21 (1.42)	24 (1.63)
	AUC	26 (0.17)	35 (0.22)		CGC	1 (0.07)	1 (0.07)
Met	AUA	**151 (1.56)**	**152 (1.6)**		CGA	**32 (2.17)**	**32 (2.17)**
	AUG	42 (0.44)	38 (0.4)		CGG	5 (0.34)	2 (0.14)
Val	GUU	**118 (1.9)**	**118 (1.9)**	Tyr	UAU	**116 (1.63)**	**114 (1.61)**
	GUC	13 (0.21)	9 (0.15)		UAC	26 (0.37)	28 (0.39)
	GUA	93 (1.49)	100 (1.61)	His	CAU	**62 (1.57)**	**63 (1.56)**
	GUG	25 (0.4)	21 (0.34)		CAC	17 (0.43)	18 (0.44)
Ser	UCU	**125 (2.67)**	**118 (2.52)**	Gln	CAA	**63 (1.7)**	**57 (1.58)**
	UCC	26 (0.55)	19 (0.41)		CAG	11 (0.3)	15 (0.42)
	UCA	80 (1.71)	88 (1.88)	Asn	AAU	**107 (1.67)**	**103 (1.65)**
	UCG	7 (0.15)	9 (0.19)		AAC	21 (0.33)	22 (0.35)
	AGU	47 (1)	54 (1.15)	Lys	AAA	**80 (1.67)**	**80 (1.67)**
	AGC	7 (0.15)	8 (0.17)		AAG	16 (0.33)	16 (0.33)
	AGA	75 (1.6)	67 (1.43)	Asp	GAU	**63 (1.62)**	**65 (1.65)**
	AGG	8 (0.17)	12 (0.26)		GAC	15 (0.38)	14 (0.35)
Pro	CCU	**80 (2.16)**	**96 (2.54)**	Glu	GAA	**74 (1.72)**	**70 (1.61)**
	CCC	17 (0.46)	5 (0.13)		GAG	12 (0.28)	17 (0.39)
	CCA	42 (1.14)	38 (1.01)	Cys	UGU	**33 (1.69)**	**34 (1.7)**
	CCG	9 (0.24)	12 (0.32)		UGC	6 (0.31)	6 (0.3)
Thr	ACU	**82 (1.95)**	**79 (1.94)**	Trp	UGA	**90 (1.64)**	**100 (1.82)**
	ACC	9 (0.21)	10 (0.25)		UGG	20 (0.36)	10 (0.18)
	ACA	72 (1.71)	66 (1.62)	*	UAA	9 (1.38)	8 (1.23)
	ACG	5 (0.12)	8 (0.2)		UAG	4 (0.62)	5 (0.77)

^a^ The higher values of preferentially used codons are in bold.

## Supplementary Materials

Supplementary materials can be found at https://www.mdpi.com/1422-0067/21/10/3545/s1. Table S1: List of nucleotide composition of *N. glans* (*Ngla*) and *N. siquijorensis* (*Nsiq*), with respect to whole genome, PCGs (considering three different codon positions), tRNA and rRNA. Figure S1: Phylogenetic relationship of *Nassarius* based on amino acid sequences of 13 mitochondrial PCGs and nucleotide sequences of 2 rRNA genes. Figure S2: Phylogenetic relationship of *Nassarius* based on the nucleotide sequences of 13 PCGs only using PhyloBayes program.
