# Supplementary material for: Complete Mitochondrial Genomes of Two Toxin-Accumulated Nassariids (Neogastropoda: Nassariidae: Nassarius) and Their Implication for Phylogeny
Source: Int J Mol Sci. 2020 May 17;21(10):3545. doi: 10.3390/ijms21103545 (PMC7278921; doi:10.3390/ijms21103545)
Supplement: Supplementary file 1 [file ijms-21-03545-s001.pdf]

Table S1. List of nucleotide composition of *N. glans* (*Ngla*) and *N. siquijorensis* (*Nsiq*), with respect to whole genome, PCGs (considering three different codon positions), tRNA and rRNA.

	**A%**		**T%**		**G%**		**C%**	
	Ngla	***Nsiq***	***Ngla***	***Nsiq***	***Ngla***	***Nsiq***	***Ngla***	***Nsiq***
Genome	30.85	30.81	39.55	39.37	15.78	15.83	13.82	13.99
PCGs	28.57	28.62	41.16	41.16	15.87	15.78	14.40	14.44
PCG1th	27.63	27.47	33.65	33.70	24.13	24.21	14.59	14.62
PCG2th	18.60	18.57	44.28	44.28	15.85	15.90	21.27	21.25
PCG3th	39.47	39.82	45.56	45.51	7.64	7.22	7.32	7.46
tRNA	33.55	33.76	35.35	34.97	16.68	16.54	14.42	14.73
rRNA	38.58	38.30	34.67	33.95	15.53	15.98	11.23	11.76

Figure captions

Figure S1. Phylogenetic relationship of *Nassarius* based on amino acid sequences of 13 mitochondrial PCGs and nucleotide sequences of 2 rRNA genes. The BI (A) and ML (B) phylograms are shown. Numbers at nodes are support values from BI (posterior probabilities) and ML (bootstrap proportions).

Figure S2. Phylogenetic relationship of *Nassarius* based on the nucleotide sequences of 13 PCGs only using PhyloBayes program. Numbers at nodes are support values from BI (posterior probabilities).

Fig. S1A

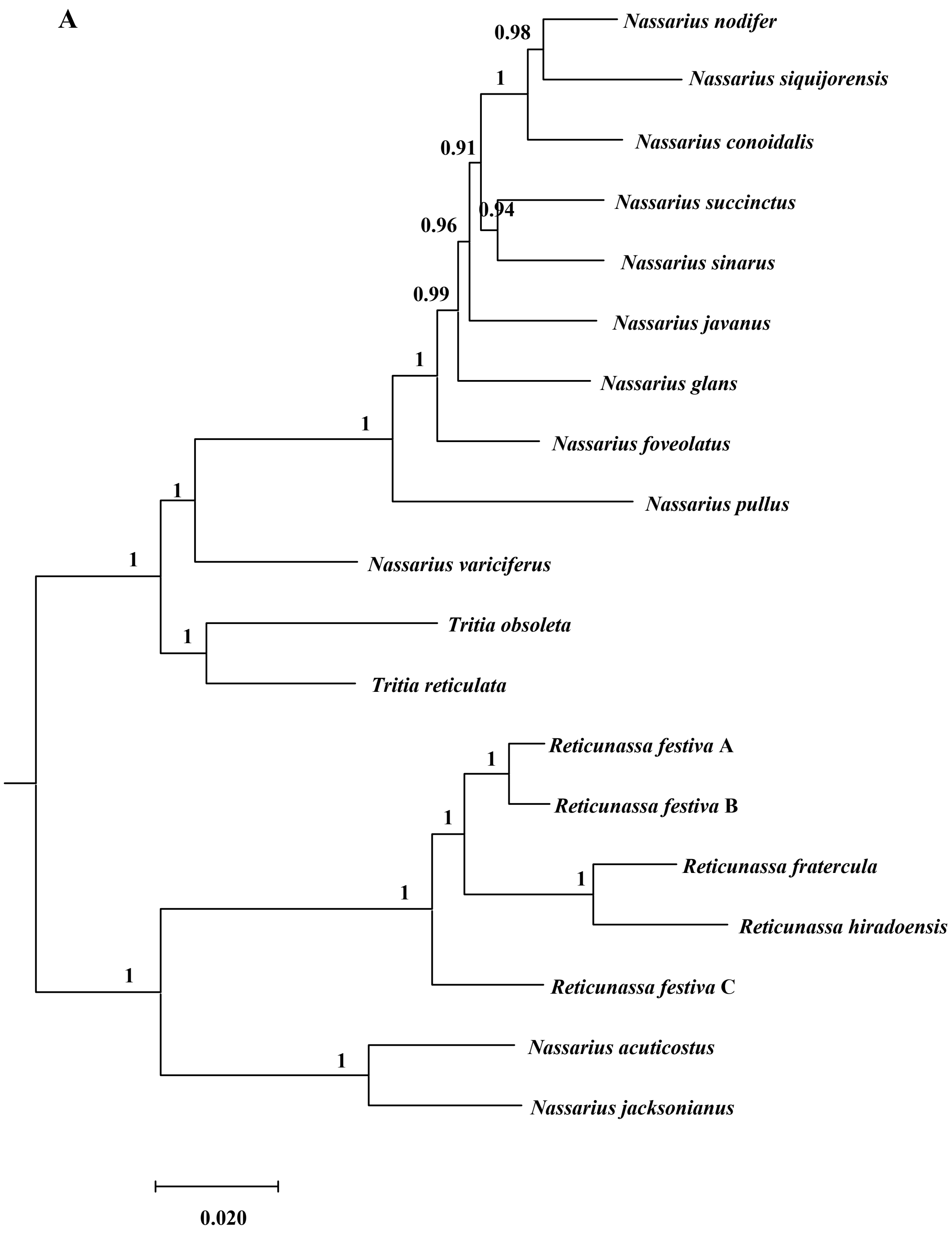
A
0.98
Nassarius nodifer
1
Nassarius siquijorensis
Nassarius conoidalis
0.91
Nassarius succinctus
0.94
0.96
Nassarius sinarus
0.99
Nassarius javanus
1
Nassarius glans
1
Nassarius foveolatus
1
Nassarius pullus
1
Nassarius variciferus
1
Tritia obsoleta
Tritia reticulata
1
Reticunassa festiva A
Reticunassa festiva B
1
Reticunassa fratercula
1
Reticunassa hiradoensis
Reticunassa festiva C
1
Nassarius acuticostus
Nassarius jacksonianus
0.020

Fig. S1B

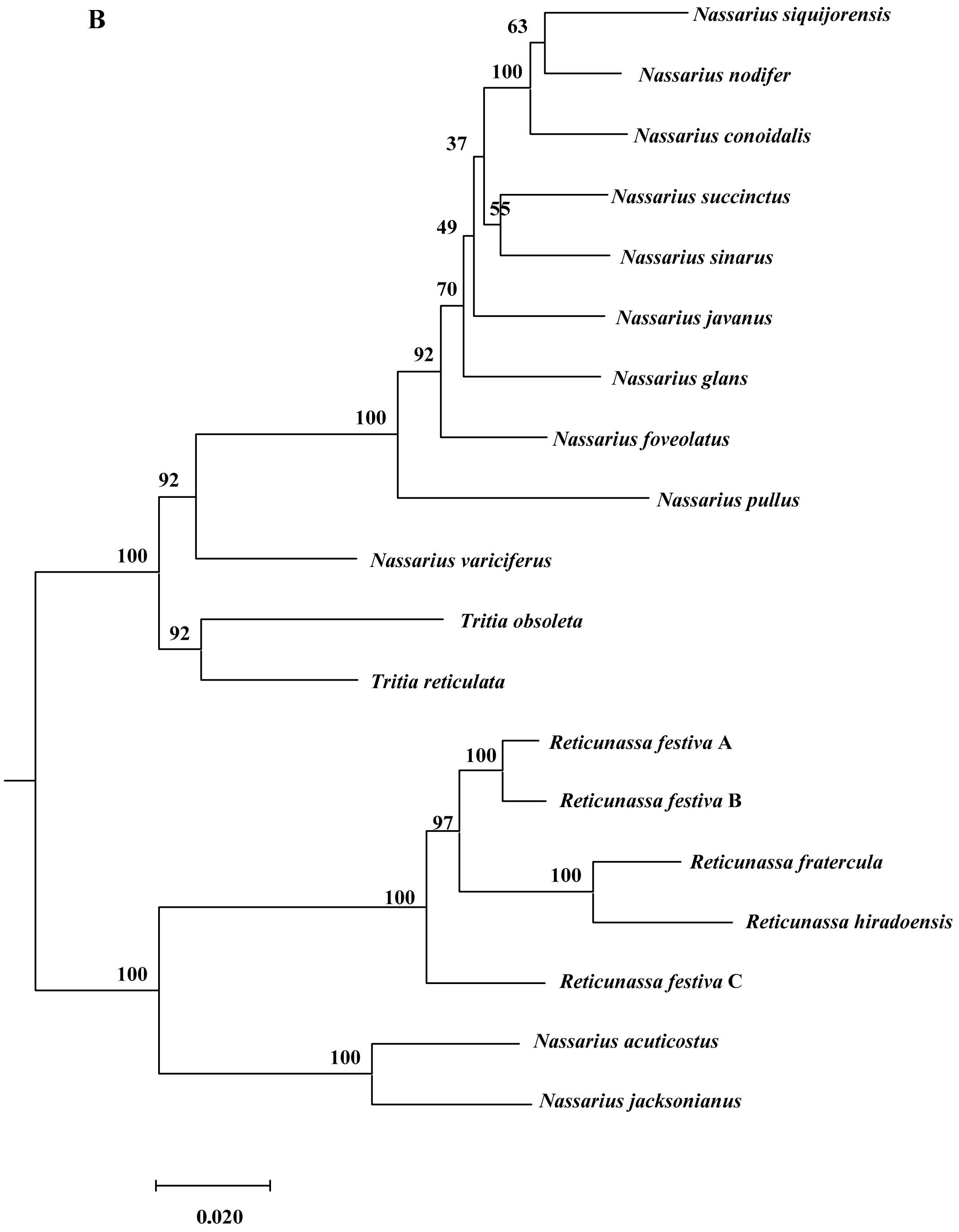
B
Nassarius siquijorensis
Nassarius nodifer
Nassarius conoidalis
Nassarius succinctus
Nassarius sinarus
Nassarius javanus
Nassarius glans
Nassarius foveolatus
Nassarius pullus
Nassarius variciferus
Tritia obsoleta
Tritia reticulata
Reticunassa festiva A
Reticunassa festiva B
Reticunassa fratercula
Reticunassa hiradoensis
Reticunassa festiva C
Nassarius acuticostus
Nassarius jacksonianus
63
100
37
55
49
70
92
100
92
100
92
100
97
100
100
100
100
0.020

Figure S2

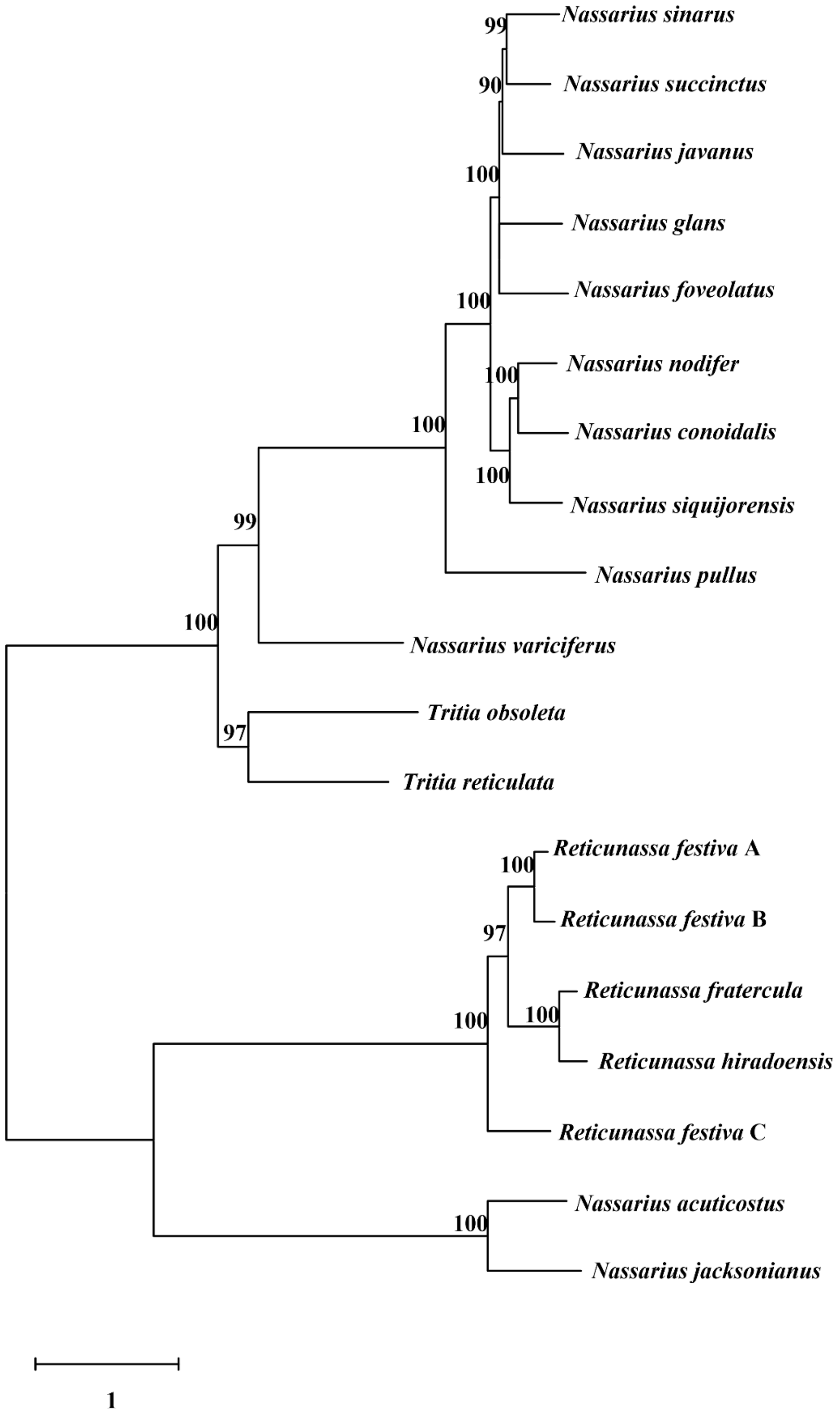
Nassarius sinarus
Nassarius succinctus
Nassarius javanus
Nassarius glans
Nassarius foveolatus
Nassarius nodifer
Nassarius conoidalis
Nassarius siquijorensis
Nassarius pullus
Nassarius variciferus
Tritia obsoleta
Tritia reticulata
Reticunassa festiva A
Reticunassa festiva B
Reticunassa fratercula
Reticunassa hiradoensis
Reticunassa festiva C
Nassarius acuticostus
Nassarius jacksonianus
99
90
100
100
100
100
100
99
100
97
100
97
100
100
100
1
